# Localizing Recent Adaptive Evolution in the Human Genome

**DOI:** 10.1371/journal.pgen.0030090

**Published:** 2007-06-01

**Authors:** Scott H Williamson, Melissa J Hubisz, Andrew G Clark, Bret A Payseur, Carlos D Bustamante, Rasmus Nielsen

**Affiliations:** 1 Department of Biological Statistics and Computational Biology, Cornell University, Ithaca, New York, United States of America; 2 Department of Molecular Biology and Genetics, Cornell University, Ithaca, New York, United States of America; 3 Center for Bioinformatics and Department of Biology, University of Copenhagen, Copenhagen, Denmark; University of Oxford, United Kingdom

## Abstract

Identifying genomic locations that have experienced selective sweeps is an important first step toward understanding the molecular basis of adaptive evolution. Using statistical methods that account for the confounding effects of population demography, recombination rate variation, and single-nucleotide polymorphism ascertainment, while also providing fine-scale estimates of the position of the selected site, we analyzed a genomic dataset of 1.2 million human single-nucleotide polymorphisms genotyped in African-American, European-American, and Chinese samples. We identify 101 regions of the human genome with very strong evidence (*p* < 10^−5^) of a recent selective sweep and where our estimate of the position of the selective sweep falls within 100 kb of a known gene. Within these regions, genes of biological interest include genes in pigmentation pathways, components of the dystrophin protein complex, clusters of olfactory receptors, genes involved in nervous system development and function, immune system genes, and heat shock genes. We also observe consistent evidence of selective sweeps in centromeric regions. In general, we find that recent adaptation is strikingly pervasive in the human genome, with as much as 10% of the genome affected by linkage to a selective sweep.

## Author Summary

A selective sweep is a single realization of adaptive evolution at the molecular level. When a selective sweep occurs, it leaves a characteristic signal in patterns of variation in genomic regions linked to the selected site; therefore, recently released population genomic datasets can be used to search for instances of molecular adaptation. Here, we present a comprehensive scan for complete selective sweeps in the human genome. Our analysis is complementary to several recent analyses that focused on partial selective sweeps, in which the adaptive mutation still segregates at intermediate frequency in the population. Consequently, our analysis identifies many genomic regions that were not previously known to have experienced natural selection, including consistent evidence of selection in centromeric regions, which is possibly the result of meiotic drive. Genes within selected regions include pigmentation candidate genes, genes of the dystrophin protein complex, and olfactory receptors. Extensive testing demonstrates that the method we use to detect selective sweeps is strikingly robust to both alternative demographic scenarios and recombination rate variation. Furthermore, the method we use provides precise estimates of the genomic position of the selected site, which greatly facilitates the fine-scale mapping of functionally significant variation in human populations.

## Introduction

Describing how natural selection shapes patterns of genetic variation within and between species is critical to a general understanding of evolution. With the advent of comparative genomic data, considerable progress has been made toward quantifying the effect of adaptive evolution on genome-wide patterns of variation between species [1–5], and the effect of weak negative selection against deleterious mutations on patterns of variation within species [1,5,6]. However, relatively little is known about the degree to which adaptive evolution affects DNA sequence polymorphism within species and what types of selection are most prevalent across the genome. Of particular interest is the effect of very recent adaptive evolution in humans. If one can localize adaptive events in the genome, then this information, along with functional knowledge of the region, speaks to the selective environment experienced by recent human populations. Another reason for the interest in genomic patterns of selection is that recent studies [3,5] have suggested a link between selected genes and factors causing inherited disease; furthermore, several established cases of recent adaptive evolution in the human genome involve mutations that confer resistance to infectious disease (e.g., [7,8]). Therefore, knowledge of the location of selected genes could aid in the effort to identify genetic variation underlying genetic diseases and infectious disease resistance. From a theoretical perspective, both the relative rate of adaptive evolution at the molecular level and the degree to which natural selection maintains polymorphism have been the subjects of intense debate in population genetics and molecular evolution [9–12]. With genome-scale polymorphism data becoming available, it is now possible to address these decades-old problems directly.

Adaptive events alter patterns of DNA polymorphism in the genomic region surrounding a beneficial allele, so population genetic methods can be used to infer selection by searching for their effects in genomic single-nucleotide polymorphism (SNP) data. Several recent studies [13–16] have taken this approach to scan the human genome for evidence of recent adaptation. These studies identify several regions of the genome that have recently experienced selection, and they suggest that adaptation is a surprisingly pervasive force in recent human evolution. However, the results of these analyses can only be considered preliminary. All of these studies have focused on the empirical distribution of a given test statistic, reasoning that loci with extreme values will be the most likely candidates for selective sweeps. This approach provides a sensible way to rank loci according to their signal of recent adaptation, but because we do not know how common selection is in the genome, the “empirical *p* value” approach does not directly test the hypothesis of selection for any individual locus, and it provides no means for quantifying how common selection is across the genome [17,18]. For instance, the null hypothesis of selective neutrality could be true for the entire genome, in which case even the most extreme values would carry no information regarding selection. Also, there are no a priori criteria available for deciding how extreme a region needs to be in order to identify selection. In short, these previous studies do not estimate their uncertainty in identifying selection. Another concern is that the statistical properties of previous methods have only been explored under the very simplest evolutionary models. Complex factors such as demographic events in the history of the population, recombination rate variation, and the biasing effects of SNP ascertainment protocols all have the potential to systematically cause false signals of natural selection, yet previous methods for identifying recent adaptation have not been thoroughly tested for their robustness to these complicating factors.

In this paper, we present a full statistical analysis of evidence for selective sweeps in the human genome using a method for detecting sweeps that has been thoroughly tested for robustness to demography and recombination rate variation, and that explicitly incorporates SNP ascertainment protocols. We apply this approach to dense genomic polymorphism data [19] with uniform SNP discovery protocols. A recent selective sweep (a bout of adaptive evolution that fixes a beneficial mutation) alters patterns of allele frequency at linked sites, eliminating variation at tightly linked loci and creating a relative excess of alleles at very low and very high frequencies at more distant loci [20–22]. Because the effect of a selective sweep will depend on the genomic distance away from the beneficial mutation, we use a statistical method (test 2 in [22]) that searches for the unique spatial pattern of allele frequencies along a chromosome that is found after a selective sweep. Essentially, the test uses a composite likelihood ratio (CLR) to compare a neutral model for the evolution of a genomic window with a selective sweep model. In the neutral null model, allele frequency probabilities are drawn from the background pattern of variation in the rest of the genome. In the selective sweep model, allele frequency probabilities are calculated using a model of a selective sweep that conditions on the background pattern of variation. Allele frequency probabilities also depend on two parameters: the genomic position of the selective sweep (*ψ*), and a compound parameter (*α*) that measures the combined effects of the strength of selection and the recombination rate between a SNP and the selected site.

Extensive simulations under a variety of evolutionary models indicate that this CLR approach is not misled by demographic events in the population's history, such as population size changes, divergence, subdivision, or migration. Furthermore, simulations indicate that this is the only available method for detecting sweeps that is not highly sensitive to assumptions about the underlying recombination rate or recombination hotspots. This lack of dependence on demography and recombination allows us to calculate *p* values for individual loci that are consistent across a wide range of selectively neutral null models. Hence, we can reliably measure our uncertainty in identifying selective sweeps, and we can obtain rough estimates of the prevalence of recent adaptation across the genome. Also, the present analysis is one of the first to fully correct for the bias introduced by SNP discovery protocols, and we account for the effects of multiple hypothesis testing using a false discovery rate approach [23,24]. The method we use provides an accurate estimate of the genomic location of the selected allele, a feature that greatly facilitates mapping of the genomic targets of natural selection. A final important difference between our analysis and previous work is that the method we use searches for the signature of “complete” selective sweeps (i.e., adaptation where the beneficial mutation has recently attained a frequency of ~100% in the population). In contrast, methods based on extended haplotype length and high linkage disequilibrium [14–16] have the most power to detect “partial” selective sweeps [15] (i.e., where the beneficial mutation has not yet spread throughout the entire population). Therefore, the two approaches are complementary, and most loci where we discover evidence for recent adaptation were not detected by previous genome-wide scans for selection or targeted candidate gene approaches.

## Results

Table 1 lists the 101 genomic locations that show very strong evidence for a recent, complete selective sweep (CLR *p* < 10^−5^), excluding locations where the estimate of sweep position was greater than 100 kb from a known gene, and excluding centromeric regions. Genomic locations with very strong evidence for a selective sweep, but not within 100 kb of a known gene, are shown in Table S1, and application of the CLR test via sliding window analyses of all autosomes are given in Table S2. Under the model of a recent and strong selective sweep, the composite likelihood estimate of the position of the selective sweep is very accurate (to within ~20 kb in regions with typical recombination rates; see [22]), so the gene nearest the estimate of sweep position is generally the best candidate as the target of selection. However, we cannot rule out the possibility that unknown functional elements or, in very gene-dense or low-recombination regions, another nearby gene might be the true target of selection.

**Table 1:**
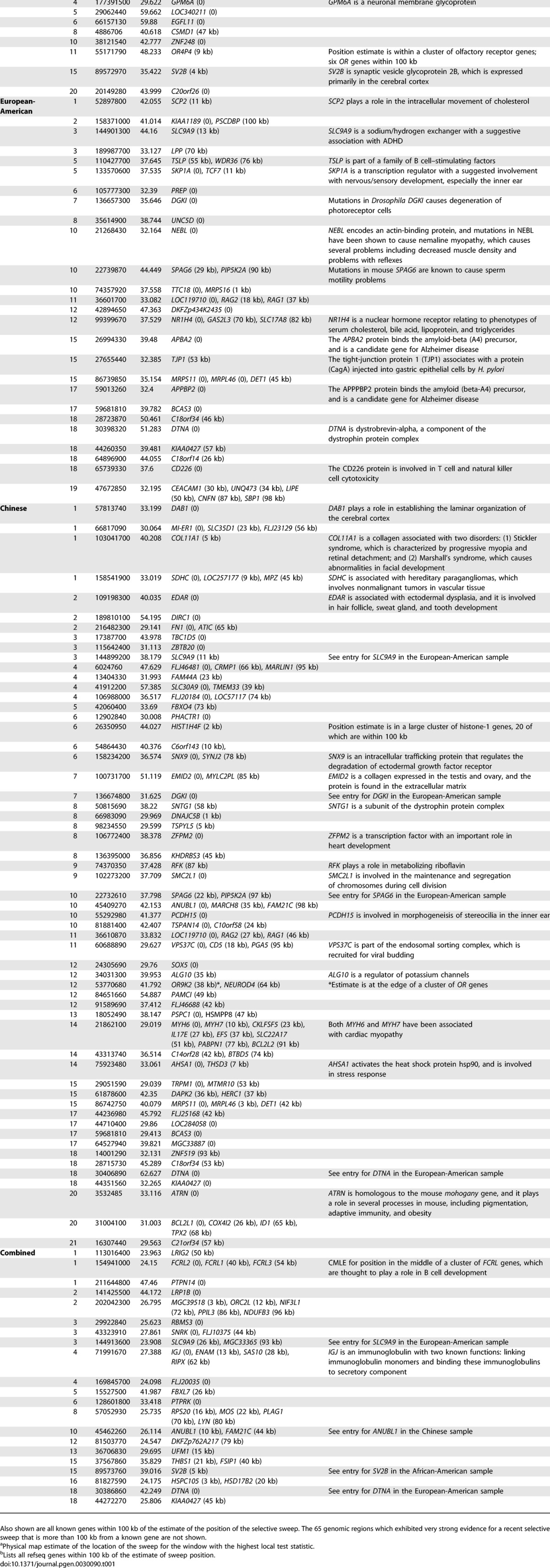
The 101 Regions of the Human Genome with the Strongest Evidence (*p* < 0.00001, CLR Test) for a Recent Selective Sweep from a Sliding Window Analysis of the Combined, African-American, European-American, and Chinese Samples

The genomic region with the strongest evidence for a recent selective sweep is in the *DTNA* gene on Chromosome 18; this location shows very strong evidence for selection in the Chinese, European-American, and combined samples. In the Chinese sample, the observed CLR statistic in this region is 62.63. In contrast, the highest CLR statistic for the Chinese population over 100,000 selectively neutral simulations is 24.34, and the 95th percentile of the simulated neutral datasets is 9.57. These simulations were performed with population bottleneck parameters that have been fit to human data [25] and with a recombination rate that is slightly less than that of the *DTNA* region. *DTNA* encodes the dystrobrevin protein, a component of the dystrophin protein complex (DPC). Aside from *DTNA*, several other genes that contribute to the DPC show evidence for recent selective sweeps (Table S3), including several syntrophin and sarcoglycan genes. The DPC primarily functions as a key structural component in the architecture of muscle tissue [26], suggesting that the selective sweeps at DPC genes may involve a muscle-related phenotype. Furthermore, several other muscle-related genes show very strong evidence for recent selective sweeps, including *NEBL* and two tightly linked, cardiac-specific myosin heavy-chain genes *(MYH6* and *MYH7).*

**Figure 1:**
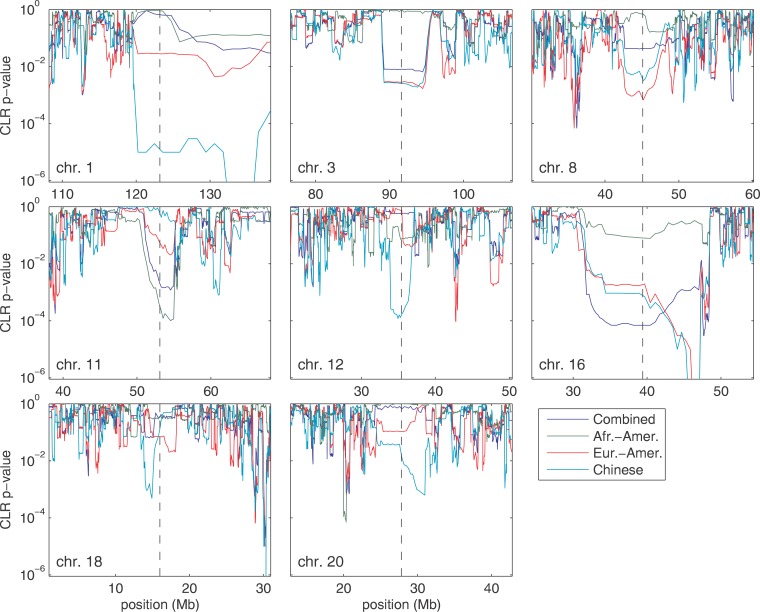
Evidence for Selective Sweeps in Centromeric Regions of Several Chromosomes, as Measured by the *p* Value of the CLR Test in Three Human Populations Vertical dashed lines indicate the positions of the centromere, and *p* values are plotted on a log scale.

One of the most conspicuous features of our genomic scan is that several centromeric regions have extreme spatial patterns of allele frequency consistent with recent selective sweeps. For instance, the region spanning the centromere of Chromosome 16 shows strong evidence of recent selection. The size of the affected area is remarkable: the combined, European-American, and Chinese samples exhibit skewed frequency spectra and very low *p* values by the CLR test over 16 Mb. Of the 17 autosomes for which we have data spanning the centromere, we observe evidence of selective sweeps in centromeric regions of Chromosomes 1, 3, 8, 11, 12, 16, 18, and 20 (Figure 1). Because the CLR test is not very sensitive to the underlying recombination rate [22], it is unlikely that this signal is an artifact of reduced recombination rates in centromeric regions. The large genomic distance over which the signature of selection extends in many of these regions complicates the identification of the selected target. However, the consistent signal of selective sweeps and the paucity of known genes in centromeric regions suggest the hypothesis that the centromeres themselves may be the functional genomic elements targeted by selection. One interesting possibility in this regard is that selection in centromeric regions may be the result of meiotic drive [27–29] (e.g., during female meiosis, any variant which even slightly decreases the probability that a chromosome segregates to a polar body will carry a huge selective advantage [30]). Also, centromeres are strong candidates for regions affecting chromosomal segregation.

**Figure 2:**
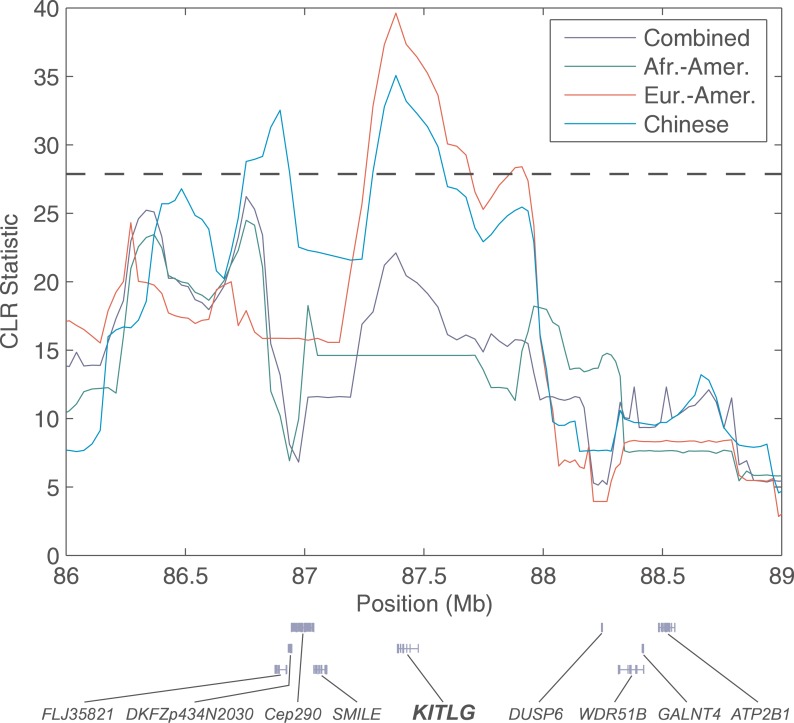
Sliding Window Analysis of the *KITLG* Region of Chromosome 12, Along with Gene Models of All refseq Genes in the Region The horizontal dashed line represents the *p* < 0.001 critical value of the population-specific CLR tests generated using a conservative estimate of the average recombination rate in the region.

Because of the time scale in which the CLR test has power to detect a selective sweep (within the last ~200,000 y), it is useful for identifying selected changes that occurred in one or more populations since the time of population divergence (the continental populations represented by the samples probably diverged within the last 100,000 years). Such population-specific selective sweeps should be evident in our analysis as a high CLR statistic and low CLR *p* value in only one of the continental groups that was sampled. Along these lines, Jablonski and Chaplin [31] suggested that global variation in skin pigmentation is due to adaptation to local environments, noting that skin pigmentation in indigenous human populations correlates very strongly with the local average intensity of UV radiation. To investigate the role of local adaptation in shaping global patterns of human skin pigmentation, we interrogate pigmentation candidate genes (Table 2) for evidence of population-specific selective sweeps. *KITLG,* which encodes a signaling molecule that stimulates melanocyte proliferation, growth, and dendricity [32], shows strong evidence for selective sweeps in the European-American and Chinese samples (Figure 2). Notably, the coding sequence of *KITLG* is 218 kb away from our estimate of the sweep position, whereas the next-nearest gene is 550 kb away, indicating that *KITLG* is the likely target of selection. Furthermore, the distance between our estimate of the sweep position and the *KITLG* coding sequence suggests the hypothesis that the selected mutation may be regulatory in nature. The presence of a selective sweep or sweeps at *KITLG,* along with experimental phenotypic effects of the gene, suggests that *KITLG* may be an important quantitative trait locus underlying variation in human skin pigmentation.

**Table 2:**
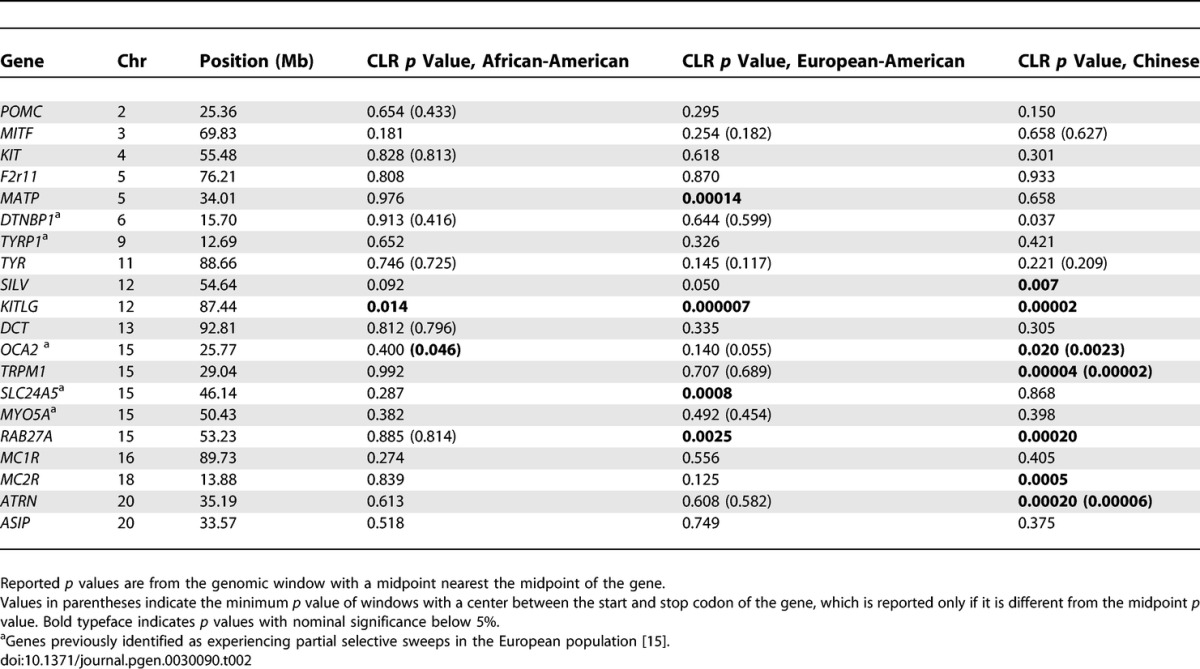
Candidate Genes for Variation in Human Skin Pigmentation and Evidence of Population-Specific Selective Sweeps

Other pigmentation candidate genes with strong evidence of population-specific selective sweeps include *RAB27A, MATP, MC2R, ATRN, TRPM1,* and *SLC24A5. SILV* and *OCA2* show marginally significant evidence for population-specific sweeps. Mouse orthologs of most of these genes carry coat color phenotypes, and *SLC24A5* was recently shown to contain a common mutation affecting skin pigmentation in humans [33]. Considered as a whole, pigmentation candidate genes are enriched for significant CLR tests. For instance, in the genome scan of the Chinese sample, pigmentation genes contain more than twice as many significant CLR tests (at the *p <* 0.01 level) compared with the expectation from the rest of the genome; this enrichment is marginally significant (χ^2^_(1)_ = 6.04, *p =* 0.007). Using a more stringent significance level for the CLR test, the enrichment of pigmentation genes becomes more pronounced (i.e., at the *p* < 0.001 level), and pigmentation genes are more than 5-fold enriched for significant tests, compared with the genomic expectation (χ^2^_(1)_ = 17.3948, *p =* 1.5 × 10^−5^). A similar pattern emerges in the European-American sample: at the CLR *p* < 0.01 level, we observe twice as many significant pigmentation genes as expected (χ^2^_(1)_ = 2.6297, *p =* 0.052), and at the *p* < 0.001 level, we observe a nearly 5-fold enrichment (χ^2^_(1)_ = 9.057, *p =* 0.0013). In a similar analysis, Voight and coworkers [15] identified a signal of partial selective sweeps in the European population for *OCA2, MYO5A, DTNBP1, TYRP1,* and *SLC24A5,* all of which are pigmentation candidate genes. Likewise, Izagirre and coworkers [34] found evidence of a partial selective sweep at *TP53B1* and *RAD50* in African populations, and at *TYRP1* and *SLC24A5* in European populations. A partial sweep occurs when the beneficial mutation has not spread throughout the entire population, whereas the CLR test is designed to detect beneficial mutations that have recently reached a frequency of 100% (complete sweeps). Thus, the two analyses should be complementary, and there is little overlap between the analyses in terms of which pigmentation genes are identified as selected in which populations. Taken together, these results indicate that population-specific selective sweeps, both partial and complete, have been common in genes in skin pigmentation pathways, suggesting that adaptation to local environments has driven the evolution of human skin pigmentation.

Several other gene categories and pathways show a striking pattern of recent adaptation. For instance, we observe evidence for a selective sweep mainly in the African-American sample in a region surrounding a cluster of olfactory receptor *(OR)* genes on Chromosome 11. Recent adaptive evolution appears to be a pervasive force among *OR* genes. Among 29 autosomal clusters of *OR* genes, 16 clusters show evidence of a selective sweep (CLR *p* < 0.05) in at least one of the populations. These findings corroborate work on adaptation in *OR* genes [35], and suggest that many changes in the human olfactory repertoire may have occurred very recently. Similarly, candidate genes for hair morphology show consistent signals of recent adaptation. Keratin-associated proteins (KRTAPs) are thought to play an important role in the shape of hair follicles, and we observe evidence for recent adaptation at four out of five clusters of *KRTAP* genes, mostly in the European-American sample. Perhaps the most surprising category of genes that show consistent evidence of recent adaptation is heat shock proteins (Table S4). Among 56 unlinked heat shock genes, 28 showed evidence of a recent selective sweep in at least one population at the *p* < 0.05 level. Several genes with functional roles in the development and function of the nervous system show very strong evidence (CLR *p* < 10^−5^) for a recent selective sweep. For example, *SV2B,* a gene encoding a synaptic vesicle protein with highest expression during brain development [36], exhibits strong evidence for a selective sweep in the African-American sample. Likewise, the protein encoded by *DAB1* plays a developmental role in the layering of neurons in the cerebral cortex and cerebellum [37], and exhibits strong evidence for a selective sweep in the Asian sample. Other nervous system genes with strong evidence for a selective sweep include two candidate genes for Alzheimer disease *(APPBP2* and *APBA2)* that bind the amyloid-beta precursor protein, two genes *(SKP1A* and *PCDH15)* with a role in sensory development, and several others with various roles in nervous system development and function *(PHACTR1, ALG10, PREP, GPM6A,* and *DGKI).*

Several analyses (e.g., [3–5]) suggest genes that play a role in immunity and pathogen response are among the most common targets of adaptive evolution. Consistent with these results, we observe very strong evidence of recent adaptation (CLR *p* < 10^−5^) within or very close to several immune system genes. These include: (1) two genes thought to play a role B-cell development *(FCRL2* and *TSLP)*; (2) two somatic recombination-activating genes *(RAG1* and *RAG2),* which help generate the diversity of immunoglobulins and T cell receptors; (3) *CD226,* a *trans*-membrane protein involved in the cytotoxicity of natural killer cells and T cells; and (4) *IGJ,* an immunoglobulin responsible for linking other immunoglobulins to each other and to the secretory component. In addition, two genes that are not part of the immune system, but which might play an important role in pathogen interactions, also show very strong evidence of a recent sweep; these are *TJP1* and *VPS37C.* The TJP1 protein associates with the CagA protein [38], which is translocated into gastric epithelial cells by the human pathogen *Helicobacter pylori.* The *TJP1*–CagA interaction is thought to play a role in the pathogenicity of *H. pylori,* and the selective sweep in the *TJP1* region suggests the hypothesis that the selected variation may have affected the pathogenic effects of H. pylori infection. The VPS37C protein is a subunit of the endosomal sorting complex, which is recruited by HIV and other viruses to promote viral budding from infected cells [39].

Several loci in the human genome have been previously identified as targets of recent adaptive evolution. Because these loci were identified using independent data and different statistical methods, they are to some extent positive controls (i.e., if selection is truly operating in these regions and if the CLR test has sufficient power, then we should observe evidence for selective sweeps at many of these loci using our approach). One such locus is the *LCT* gene on Chromosome 2. Numerous studies have identified evidence for one or more functional polymorphsims in *LCT* that affect lactose metabolism in adults [40,41], and Bersaglieri and coworkers [42] found that very recent positive selection in European populations has strongly affected the frequency of this polymorphism. Concordantly, we observe evidence for a selective sweep in the European-American sample (CLR *p* = 0.012), but not the other samples. Notably, the proposed beneficial mutation in *LCT,* the lactase persistence allele, is not completely fixed in European populations; rather, its frequency is 77% [42]. Even though the CLR test considers a model of a complete selective sweep in which the beneficial allele reaches a frequency of 100%, the significant result at *LCT* suggests that the CLR test has at least some power to detect recent adaptive events that deviate from the assumptions of the complete sweep model. The *HFE* gene on Chromosome 6 is another locus for which previous work suggests a selective sweep [43]. For the genomic window centered on *HFE,* we find significant evidence for a selective sweep in the vicinity of *HFE* in the Chinese (*p* = 0.00006), European-American (*p* = 0.002), and combined (*p* = 0.0006) samples. *HFE* contains a relatively high-frequency recessive mutation, C282Y, which causes hereditary hemochromatosis [44], an iron-overload disorder. Although positive selection is thought to operate somewhere in the vicinity of *HFE,* it is unknown whether the C282Y mutation attained high frequency through selection directly (positive selection on C282Y itself) or indirectly (positive selection on a nearby beneficial mutation associated with C282Y). Our composite likelihood estimate of the position of the selective sweep is within a cluster of histone genes, 150 kb away from *HFE,* suggesting that C282Y may have attained high frequency through association with a nearby beneficial allele. If this hypothesis of C282Y rising to high frequency indirectly is correct, then it carries the interesting implication that populations experiencing selective sweeps may sometimes incur indirect costs: occasionally, selective sweeps may carry tightly linked, initially rare, deleterious, and potentially disease-causing variation to relatively high frequencies [45]. Essentially, a recent selective sweep may have a localized effect in the genome similar to a population bottleneck (i.e., a sweep is somewhat analogous to a genomically localized reduction in effective population size), and deleterious disease alleles in these regions may obtain observable frequency by chance in this situation. Other regions where previous research has suggested positive selection, and the signal is confirmed by our analysis, include the cluster of *ADH* genes on Chromosome 4 [46], which show evidence for a recent sweep only in the Chinese sample (CLR *p* = 0.00015), and the opioid receptor *PDYN* [47], which also shows evidence of a selective sweep only in the Chinese sample (CLR *p* = 0.002). Loci that have been previously identified as targets of recent or ongoing selective sweeps, but do not show evidence for a selective sweep in the present analysis, include *MMP3* [48], *CD40LG* [8], *CCR5* [7], *ASPM* [49], and *MCPH* [50]. Like *LCT,* previous work indicates a partial selective sweep at these loci, and in all of the above cases, the frequency of the putatively beneficial allele is relatively low (between 10% and 70%). Because these loci are thought to deviate more strongly from the complete sweep model, the CLR test probably does not have adequate power to detect selection at these loci.

Another means of validation for our genomic scan is to compare the spatial distribution of evidence for selection along chromosomes with the distribution of known functional elements in the genome (i.e., if a large proportion of positive tests are false positives, then one would not expect positive tests to be associated with functional elements). For example, Voight and coworkers [15] found that genic regions of chromosomes are strongly enriched for extreme values of the integrated extended haplotype homozygosity statistic, an observation that is not readily explainable by factors that can cause a false signal of selection, such as demography or ascertainment bias. Using a similar approach, we tested regions surrounding known genes for an enrichment of significant CLR tests. We used a contingency table approach to test for enrichment (i.e., we compared the proportion of significant tests in windows nearest the center of known genes to the proportion of significant tests in the remainder of the genome). The results of these analyses are given in Table S5. Notably, in the European-American and Chinese samples, we observe a strong excess of significant tests in genic regions, and this signal becomes stronger as the significance level applied to the CLR test becomes more stringent. For example, in the European-American sample at a significance level of *p* < 0.001, we observe 40% more significant tests than expected at gene centers, based on the total number of significant tests and the total number of windows at gene centers. Because centromeric regions have strong evidence of selection and low gene density, this signal becomes even stronger if centromeric regions are excluded. We conclude, therefore, that extreme values of the CLR statistic are strongly associated with genic regions of chromosomes, and this association has two important implications. First, it further corroborates the results of our genomic scan for selective sweeps, as this association is not predicted if a high proportion of significant tests are false positives. Second, the association between genes and selection in this paper and in the Voight et al. [15] study suggests that the empirical follow-up to genomic scans for selection will be at least somewhat experimentally tractable. Identifying beneficial mutations and determining their phenotypic effects will be much easier if the beneficial mutation is within a known gene.

Another interesting comparison is the contrast between our analysis and previously published genomic scans for selective sweeps. This comparison does not necessarily provide a means of validating ours or previous analyses, as the statistics used in the different genomic scans may be correlated even under selective neutrality, and the statistics have power to detect different types of selective sweeps. However, the comparison does provide a general sense of the consistency of population genetic methods for identifying selective sweeps from genomic variation data. Table S6 gives the CLR statistics and *p* values for the most extreme regions of the genome identified in [16] using two different approaches: population differentiation (Table 9 in [16]) and extended haplotype homozygosity [8] (Table 10 in [16]). In the Chinese sample, genes containing nonsynonymous SNPs that exhibit high levels of population differentiation in the Hapmap data [16] are enriched for CLR tests significant at the *p* < 0.01 level (χ^2^_(1)_ = 10.6; *p* = 0.0011). Similarly, genomic regions with the most extreme patterns of extended haplotype homozygosity in the Hapmap data [16] also have more significant CLR tests than would be expected if the two statistics were statistically independent. However, even among the most extreme regions of the genome in the Hapmap analysis, the CLR analysis does not always show evidence of a selective sweep. This inconsistency is likely the result of differential power of the alternative approaches in detecting different types of selection. For example, considering that extended haplotype approaches [8] have the most power to detect partial selective sweeps [15], it would not be surprising if the most extreme regions of the genome by these approaches were the result of a partial sweep. Furthermore, the CLR approach probably has limited power to detect this type of selection because it does not leave a population genetic signature similar to that of a complete sweep. In conclusion, it is encouraging that the CLR test is not independent of other statistics, which suggests some consistency among genomic scans for selective sweeps. However, it is also encouraging that the CLR test is not completely correlated with other approaches; if it were, then we would not have uncovered any previously unknown selective sweeps in this analysis.

**Figure 3:**
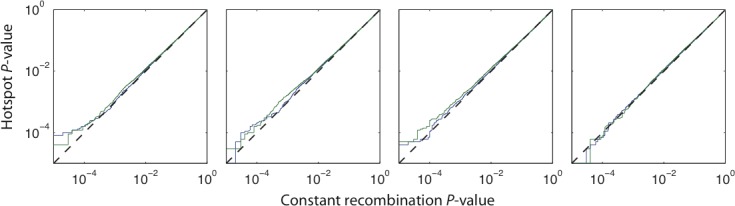
A Comparison of *p* Values of the CLR Test, Calculated from Simulations of Models Assuming a Constant Recombination Rate and Models That Include Recombination Hotspots (A) The combined sample. (B) The African-American sample. (C) The European-American sample. (D) The Chinese sample. *p* Values are highly consistent between constant recombination and hotspot models, indicating that the CLR test is robust to recombination rate variation. Note that both axes are on a log scale.

In addition to the statistical exploration of the CLR test by Nielsen et al. [22], we performed extensive neutral simulations to determine how robust the CLR approach is to both recombination rate variation and complex demography. Recent work suggests that recombination rate variation is a pervasive feature of the human genome, and most recombination events occur in recombination hotspots [51,52]. To investigate how recombination rate variation might affect our analysis, we performed coalescent simulations with recombination hotspots, as well as SNP ascertainment, missing data, and different demographic scenarios. Recombination hotspots were represented as randomly spaced 5 kb fragments with an average distance between hotspots of 50 kb, and within the hotspot, the recombination rate was assumed to be 8-fold higher than the background rate. Figure 3 shows a comparison of *p* values calculated from a constant recombination model and a hotspot model with an equal average recombination rate. Recombination rate variation appears to have no effect on the null distribution of the CLR statistic, and *p* values calculated under the hotspot and constant recombination models are strikingly consistent. We observe some minor differences in *p* values calculated for very extreme test statistics (*p <* 10^−4^), but these differences are readily explainable by Monte Carlo error in the estimation of *p* values via simulation.

**Figure 4:**
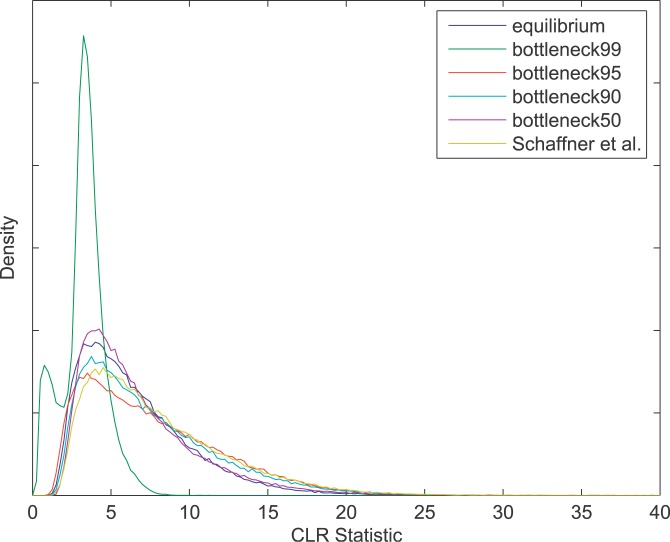
The Null Distribution of the CLR Statistic in a Non-African Population for Non-African Bottleneck Models of Varying Strength, As Well As the Complex Schaffner Model

We also performed simulations under a variety of demographic models beyond those considered by Nielsen et al. [22] in order to more fully explore the robustness of the CLR test to complex population demography. In particular, we investigated how the strength of the population bottlenecks experienced by non-African populations affects the null distribution of the CLR statistic. We simulated data under population bottlenecks with a constant duration and varying severity, with the temporary reduction in population size ranging from 50% to 99% only for non-African populations. Surprisingly, the null distribution of the CLR statistic is shifted toward lower values under the strong bottleneck model (99% reduction) compared with the equilibrium model (Figure 4), and the variance in the CLR statistic is much lower. This result indicates that, if the strong bottleneck model accurately reflects history, but we use the equilibrium model (random mating, constant population size) to obtain *p* values of the CLR test, our results will be strongly conservative. These surprising results for the strong bottleneck model can be explained by a coalescent argument: with a strong and recent bottleneck, the vast majority of the coalescences and the most recent common ancestor of the sample typically occur during the bottleneck, which reduces the stochasticity due to the ancestral process. This reduced stochasticity results in less variation in the site-frequency spectrum (SFS) across the genome and, consequently, less extreme CLR statistics. Under a weak bottleneck (50% reduction), the null distribution of the CLR statistic is nearly unaffected. Intermediate-strength bottlenecks (90%–95% reduction) cause the most problems: compared with the equilibrium model, the CLR statistic shows slightly more variation under intermediate bottlenecks, and the upper tail of the null distribution is slightly heavier. Similar to the case of an intermediate bottleneck model, the complex model approximated by Schaffner et al. [53] results in slightly more variation in the CLR statistic with a heavier upper tail. Therefore, the equilibrium neutral model will be somewhat anticonservative when applied to a population that has experienced an intermediate bottleneck or multiple weak bottlenecks, as in the case of the Schaffner et al. [52] model. However, compared with the effect of demography on standard methods for detecting selection, the CLR approach is very robust to even the most extreme demographic effects. The robustness of the CLR approach to demographic effects is reflected in the general consistency of *p* values obtained under alternative demographic models (Figure S1).

**Figure 5:**
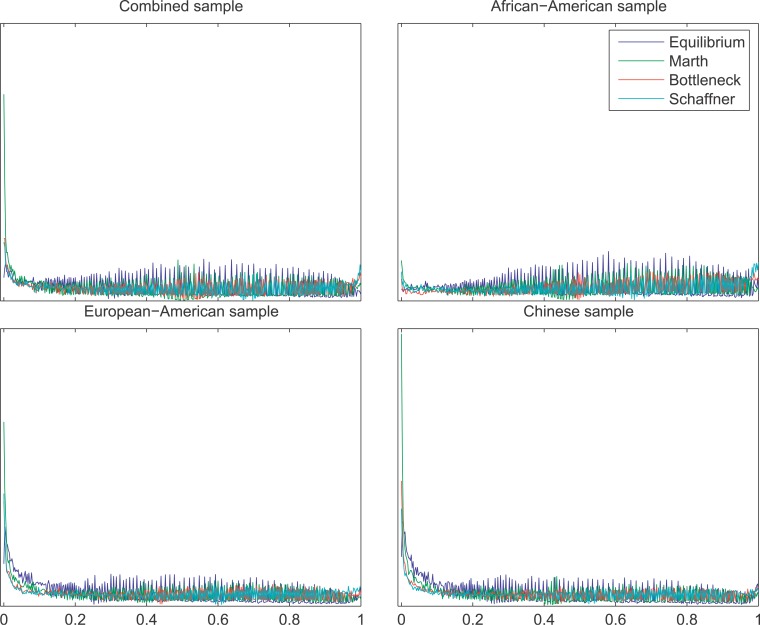
The Distribution of *p* Values for the CLR Test of a Selective Sweep

**Figure 6:**
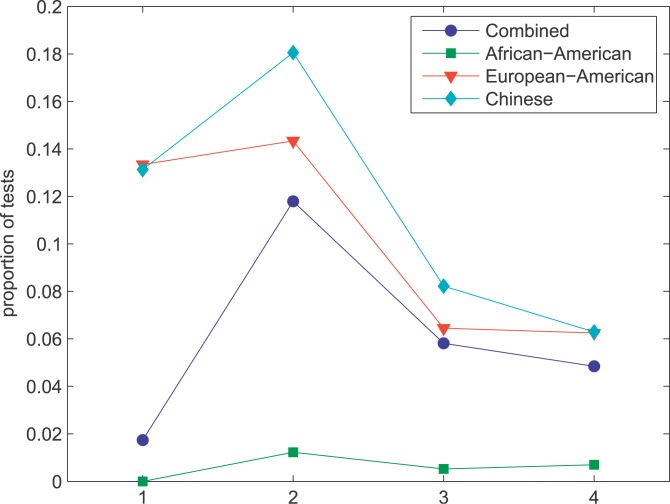
The Fraction of Tests for Which the Null Hypothesis Is False, Estimated Using a FDR Procedure and Shown for Four Alternative Evolutionary Models (1) The equilibrium, random mating, neutral model. (2) The Marth et al. [25] bottleneck and growth model. (3) The most conservative non-African bottleneck model. (4) The complex demographic and recombination model calibrated by Schaffner et al. [50].

False discovery rate (FDR) methods [23,24] use the distribution of *p* values among tests to correct for multiple hypothesis testing, providing an estimate of the probability that the null hypothesis is true for any particular test (the *q* value). The distribution of *p* values for the different windows is shown in Figure 5. In the Chinese and European-American samples, the distribution shows a strong excess of tests with very low *p* values from the CLR test, suggesting that the null hypothesis is false for many of these windows. In addition to correcting for multiple testing, FDR methods estimate the number of tests in which the null hypothesis is false (*m_1_*). In the case of genomic scans for natural selection, *m_1_* is itself a parameter of interest, because it provides a rough indication of what proportion of the genome is affected by selective sweeps at linked sites. FDR estimates of the proportion of tests where the null hypothesis is false (*m_1_/m*) is shown in Figure 6, using several alternative demographic models to obtain *p* values. All alternative models indicate that recent selective sweeps have been a pervasive force in the human genome, with ~10% of the genome affected by selective sweeps in the European-American and Chinese samples, ~1% in the African-American sample, and ~5% in the combined sample.

The FDR estimates of *m_1_* suggest that recent adaptation has had a strong effect on genome-wide patterns of nucleotide variation, to the point that a considerable fraction of the genome is evolving nonneutrally. However, this conclusion should be considered preliminary: *m_1_* is a very rough measure of the pervasiveness of selective sweeps, and estimates of the proportion of the genome affected by a sweep will of course depend strongly on what is meant by “affected.” In our case, this means that selection has altered patterns of variation in the window sufficiently to drive the *p* value of the CLR test below ~0.05. The ability of selection to alter variation in a window will depend very much on the strength of selection, the genomic distance away from the beneficial mutation, the age of the selective event, and the type of selection. Fully describing the genomic effects of linked selection and estimating the number of selective events will require fitting a model of multiple selective events to the entire genome (perhaps including complete selective sweeps of varying age, different types of balancing selection, partial selective sweeps, and “soft” sweeps starting from standing variation), rather than fitting a model of a single selective sweep to a small window of the genome for a number of different windows. The primary utility of the present analysis lies in the fine-scale identification of individual loci that have experienced selection, which greatly facilitates the investigation of what human phenotypes have been affected by adaptation, and what forces in the environment have driven recent human evolution.

## Discussion

Here we have presented a comprehensive scan for selective sweeps across the human genome. Several general patterns emerge from the analysis. We find much more evidence for selective sweeps in Chinese and European-American populations than in the African-American population. This result is consistent with the hypothesis that, as anatomically modern humans migrated out of sub-Saharan Africa, the novel environments they encountered imposed new selective pressures, which in turn led to an increased rate of population-specific selective sweeps [54–56]. However, a caveat should be considered when interpreting the differences between African-American and non-African populations: the statistical power to detect selective sweeps is likely to be much lower in the African-American sample. Because the CLR test is based on a complete sweep model, the recent admixture of African and European lineages in the African-American population probably weakens the signal of Africa-specific selective sweeps. If a complete selective sweep occurred in African populations after the divergence of European populations, then the beneficial allele, and corresponding haplotypes, would not be fixed in the African-American sample. In other words, admixture is expected to fundamentally alter the molecular signature of a selective sweep, and it is therefore unsurprising that our results for the African-American sample are distinctly different from those of the European-American and Chinese samples. Another factor to consider is the extensive subdivision among African populations [57]. Subdivision within Africa may have allowed, or may have been driven by, adaptation to local environments within Africa. This sort of selection may not be evident in the African-American sample, which represents a nonrandom, continent-wide sampling of African lineages with some admixture of European lineages [58]. Subdivision within Africa may add further complications to the effect of admixture on the power of the CLR test (i.e., perhaps the proper demographic history of the African-American population includes the admixture of several diverged African populations), followed by large-scale (20%, from [59]) admixture with European populations. For example, in this demographic scenario, if a selective sweep occurred within Africa in a source population for the African-American population, the molecular signature of this sweep would be obscured by the admixture among African populations during the founding of the African-American population, and the signature would further be eroded by subsequent admixture with the European population. Considering that numerous factors suggest that selective sweeps will be much more difficult to detect in the African-American sample, compared with the non-African populations, it is premature to conclude that the rate of adaptation has increased in non-African populations.

Another general pattern that emerges from our analysis is that we observe more evidence for selective sweeps within subpopulations, compared with the cosmopolitan sample. This result suggests that adaptation to local environments has been an important force in recent human evolution. The relevance of local adaptation might be predicted considering the extensive range expansions in recent human history, and the tremendous diversity of environments inhabited by indigenous human populations. However, the notable discrepancy between local and cosmopolitan sweeps is also difficult to interpret due to potential differences in the statistical power to detect different types of selective events. For example, if the power to detect sweeps were much greater in the local samples compared with the cosmopolitan sample, then one would expect to observe results similar to ours, even if the true number of local and cosmopolitan sweeps were equal. Fully evaluating the relative importance of localized and worldwide selective sweeps will require a detailed study of the statistical power to detect these types of sweeps under reasonable models of human demographic history.

In order to correct for the confounding effects of demographic history, we use a test [22] that compares allele frequencies in regions of the genome to the background pattern of variation. Simulations of a number of demographic models indicate that the methods are fairly robust to a wide variety of demographic histories; therefore, complex demography should not increase the rate of false positives, but we cannot rule out the possibility that some complicated demographic scenarios could lead to an aberrant signal of selection. Even so, if selective sweeps have affected some regions of the human genome, we feel that the regions that we have identified with extreme frequency spectra are the best candidates for future studies. Another alternative explanation of the results of the CLR test is that weak negative selection operating on the SNPs themselves could locally skew allele frequencies toward rare alleles in a manner that could mimic a selective sweep. Although we cannot rule out this explanation, several factors suggest that localized weak selection does not have a systematic effect on our results. First, the vast majority of SNPs are in genomic regions with no known function (99.2% are noncoding). Second, in most of the regions where we identify selective sweeps, the sweep is population-specific, an observation that is difficult to explain with weak negative selection. And third, we observe greater evidence for selective sweeps in non-African populations than in the African-American sample. If weak negative selection were the root cause for these deviations from neutrality, then one would expect a greater signal in the African-American sample because of the larger effective population size in African populations.

The approach we have taken here—detecting complete selective sweeps by their effects on variation at linked sites—is complementary to previous divergence-based approaches [1–5] characterizing adaptive evolution across the human genome. For instance, divergence-based approaches have been limited to detecting adaptive changes that have occurred via recurrent amino acid substitutions within a gene, whereas the present approach is capable of detecting adaptive changes at all functional genomic categories. The two approaches also differ in the time scale over which selection is detectable. Divergence-based approaches detect molecular adaptation that has occurred at any time on the lineage separating humans and chimps. Linked selection approaches, in contrast, are time-specific, detecting ongoing or very recent (within the last ~200,000 years) selection. Linked selection approaches are also much more amenable to investigating the adaptation of subpopulations to local environments at the molecular level. Given the complementary nature of divergence-based and linked selection methods, the present analysis fills in some of the gaps in our knowledge of human adaptive evolution. The challenge now is to use information about the genomic location of selective sweeps, in combination with the tools of functional genomics and knowledge of human ecology, to identify the traits that have been affected by recent adaptation and the selective forces that have shaped human populations.

## Materials and Methods

### Statistics.

To correct for the confounding effect of demography, the CLR test of a selective sweep compares the SFS of a small region of the genome (a “window”) to the SFS of the rest of the genome. The CLR test calculates the composite likelihood of the data in a window for two models: (1) a model which predicts the probability of SNP frequencies using the genomic background SFS; and (2) a model of a very recent selective sweep. The composite likelihood in the sweep model is independent of demography because the SNP frequencies among lineages that were present before the sweep are predicted using the genomic background SFS. In essence, the CLR test works by considering the spatial pattern of allele frequencies along the genomic sequence, as predicted by a selective sweep model given the background pattern of variation. In an investigation of the statistical properties of methods for detecting selective sweeps, Nielsen et al. [22] demonstrate that, among several statistical tests for detecting selective sweeps, the CLR test is the most powerful and is the most robust to demography and the underlying recombination rate. The CLR test can be applied to either the SFS of the entire sample or to population-specific subsets of the data, enabling the detection of geographically restricted selective sweeps and balancing selection. For population-specific tests, we incorporate SNPs that are variable in the combined sample, but invariable within the subpopulation (i.e., the SFS describes the number of SNPs with minor allele counts of *I =* 0,1,2…*n*/2). The inclusion of invariable SNPs may significantly increase power to detect selective sweeps because, if a population-specific sweep has occurred recently, then one expects a strong excess of invariable SNPs within the population. By using SNPs that are invariable within a subpopulation, but variable in the combined sample, our methods should be robust to mutation rate heterogeneity across the genome, which would not be true if we included all invariable sites. A full description of the tests and an exploration of their statistical properties can be found in Nielsen et al. [22].

Because allele frequencies of linked SNPs are not statistically independent, we determine the null (selectively neutral) distributions of all test statistics using coalescent simulations [60]. For data analysis, we define genomic windows based on the number of SNPs in the window; therefore, we condition on an equal number of SNPs being present in our simulated datasets. Defining windows based on the number of SNPs makes the procedure robust to both mutation rate heterogeneity and the increased variance in regional nucleotide diversity caused by nonstandard demographies such as bottlenecks (K. Thornton, personal communication). To address the effect of SNP ascertainment, we incorporate the ascertainment scheme into our simulations by simulating the genealogy of both the genotyping sample and the sample in which the SNP was discovered, and keeping only those SNPs that are variable in the discovery sample. For each SNP, the discovery sample size was determined by a random draw from the empirical distribution of discovery sample sizes, which was provided by Perlegen Sciences (http://www.perlegen.com). We incorporate ascertainment into the simulations, rather than applying an explicit ascertainment correction [61,62], because the cosmopolitan discovery sample is computationally expensive to correct for in population-specific genotyping samples. The Monte Carlo approach to correcting for SNP ascertainment is greatly simplified by the uniform SNP discovery protocol used by Perlegen; for datasets with variable SNP ascertainment, such as the hapmap SNPs [16], it would be necessary to also model the autocorrelation of ascertainment along the chromosomes. Each iteration consisted of simulating a sample with a fixed number of ascertained SNPs, dividing the sample into African-American, European-American, and Chinese samples, then calculating the combined and population-specific CLR statistics. This procedure was repeated 10^5^ times. Nielsen et al. [22] found that, among a variety of demographic models that have been fitted to human data, the equilibrium neutral model (random mating, constant population size) provides the most conservative critical values for the CLR test; therefore, all reported *p* values are from simulations of the standard neutral model. Finally, we incorporate SNPs with missing data by calculating the tests using SNP allele frequencies from a subsample of the data, summing over all possible allele frequencies in the subsample [25,62]. For the population-specific tests, the subsample size was set to *n* = 44 chromosomes, and for the combined test, it was set to *n* = 132. SNPs that did not have at least 44 chromosomes successfully genotyped in the African-American, European-American, and Chinese samples were excluded from further analysis. The correction for missing data was incorporated into the simulations of the CLR null distribution, and data was missing in the simulated data sets by randomly drawing the sample size for each SNP according to the empirical distribution of sample sizes.

The CLR statistic is weakly dependent on the underlying recombination rate: the test becomes somewhat more conservative if the assumed recombination rate is less than the true rate, and slightly anticonservative if the assumed rate is greater than the true rate. It is necessary to account for this weak dependence because: (1) recombination rates are known to vary considerably across the genome [63]; and (2) we base the size of our genomic windows on a fixed number of contiguous SNPs, so that the size of the window in base pairs will vary with SNP density. To address these issues, we estimate the recombination rate for each window of the genome based on the size of the window and genetic map estimates [63] of the local recombination rate. Then, to make the tests more conservative, we downwardly bias our estimates by a factor of five. We have simulated the null distributions of all test statistics for regional recombination rates of *r* = 0, 10^−5^, 3 × 10^−5^, 10^−4^, 3 × 10^−4^, and 10^−3^. To estimate the *p* value for each genomic window, we use our downwardly biased estimates of *r* to interpolate between *p* values calculated from the simulated null distributions with different *r*.

To account for multiple hypothesis testing, we apply FDR methods [23] that are specifically designed for genomic analyses [24]. FDR methods use the distribution of *p* values to estimate the number of tests in which the null hypothesis is false (*m_1_*), and the probability that the null hypothesis is true for any particular test (the *q* value). One modification to the approach outlined by Storey and Tishirani [24] is the method we use for selecting the tuning parameter, *λ*. First, we represent the distribution of *p* values using a histogram of 500 bins. Next, we smooth the distribution by calculating the average density of the distribution in a window surrounding a particular *p* value. Let *b* be the number of bins in the window, *a*(*P*) be the average density around *P*, and *w* be the width of the bins. Then we select the tuning parameter *λ* as the minimum *P* for which the following relation holds: [*a*(*P*) − *a*(*P* + *wb*)] / *a*(*P* + *wb*) ≤ ɛ. For the CLR test, *b* was set to 12, and *ɛ* was set to 0.1. In essence, we use this procedure to estimate the point at which the distribution of *p* values flattens out. The procedure was used because the CLR test was designed to be conservative; therefore, one expects the distribution of *p* values to be skewed somewhat toward *p* = 1. Standard methods, such as splines [26], assume the distribution of *p* values is flat near *p* = 1.

### Data.

We obtained allele frequency data for the Perlegen SNPs [19] from the Perlegen genotype browser website (http://genome.perlegen.com/browser/download.html), and ascertainment information was obtained directly from Perlegen Sciences. We limited the analysis to those SNPs that were discovered by Perlegen's chip-based resequencing in a worldwide sample of 24 individuals [64], including African-Americans, European-Americans, Native-Americans, and Asian-Americans. For analysis, we take a sliding window approach to scan the entire genome for evidence of selective sweeps and balancing selection. For a genomic window of 200 contiguous SNPs (on average ~500 kb), we perform the CLR test on the SFS of the combined sample (African-American + European-American + Chinese) and on the SFS of each of the individual populations. The values of all test statistics, corresponding significance levels, maximum likelihood estimates of the position of the sweep, and an estimate of the composite parameter *α* are then recorded along with the genomic position of the center of the window. We repeat this procedure for every tenth window of 200 SNPs across all autosomes. Chromosomal positions of genes and genetic map estimates of local recombination rates were retrieved using the July 2003 build of the human genome on the University of California Santa Cruz (UCSC) table browser [65]. A list of refseq genes mapped on to the same genomic build as the Perlegen SNPs is available either from the UCSC table browser or by request from the corresponding author.

## Supporting Information

Figure S1 A Comparison of *p* Values Calculated from the Equilibrium Neutral Model with *p* Values Calculated from Alternative Neutral Null Models Curves above the diagonal dashed lines indicate that the equilibrium model is anticonservative relative to the alternative null, and curves below the dashed line indicate that the equilibrium model conservatively identifies selection. The close correspondence between the curves and the diagonal dashed lines indicates that *p* values are largely consistent across alternative neutral null models, and demographic history does not systematically mislead the CLR approach.
(47 KB PDF)

Table S1 The 63 Genomic Regions with Strong Evidence for a Recent Selective Sweep (*p* < 0.00001, CLR test), but where the Estimate of the Position of the Beneficial Allele Is Not within 100 kb of the Coding Sequence of a Known Gene (111 KB DOC)

Table S2 A Genomic Scan for Selective Sweeps Using the CLR Test and a Sliding Window Approach Each row contains the results of the CLR test for a 200 SNP window of the genome. Columns represent (1) chromosome; (2) position of the center of the window; (3) CLR statistic for the combined sample; (4) maximum composite likelihood estimate of sweep position in the combined sample; (5) CLR *p* value for the combined sample; (6) CLR statistic for the African-American sample; (7) maximum composite likelihood estimate of sweep position in the African-American sample; (8) CLR *p* value for the African-American sample; (9) CLR statistic for the European-American sample; (10) maximum composite likelihood estimate of sweep position in the European-American sample; (11) CLR *p* value for the European-American sample; (12) CLR statistic for the Chinese sample; (13) maximum composite likelihood estimate of sweep position in the Chinese sample; (14) CLR *p* value for the Chinese sample.
(12 MB TXT)

Table S3 Evidence of Selective Sweeps at Genes Involved in the Dystrophin Protein Complex *p* values are from the test of the genomic window nearest the midpoint of the gene, and values in parentheses represent the minimum *p* value for all windows within the gene, which is reported if different from the midpoint *p* value.
(71 KB DOC)

Table S4 Evidence of Selective Sweeps at Heat Shock Genes *p* values are from the test of the genomic window nearest the midpoint of the gene.
(147 KB DOC)

Table S5 Contingency Table Analyses for Enrichment of Significant Results in Windows Nearest the Midpoint of Known Genes, Compared with the Remainder of the Genome Different rows repeat the analysis for different CLR test significance levels (indicated in parentheses) and for different population samples. For the CLR test in the European-American and Chinese samples, we observe a highly significant enrichment of CLR tests that reject the null at gene centers, and this signal becomes stronger with more stringent significance levels.
(74 KB DOC)

Table S6 Evidence of a Selective Sweep by the CLR Test in the Most Extreme Genomic Regions Identified by Other Methods in the Hapmap Analysis Values in parentheses indicate *p* values of the CLR statistic.
(99 KB DOC)

## Abbreviations

CLR: composite likelihood ratio
DPC: dystrophin protein complex
FDR: false discovery rate
*OR*: olfactory receptor gene
SFS: site-frequency spectrum
SNP: single-nucleotide polymorphism
