# Supplementary material for: Localizing Recent Adaptive Evolution in the Human Genome
Source: PLoS Genet. 2007 Jun 1;3(6):e90. doi: 10.1371/journal.pgen.0030090 (PMC1885279; doi:10.1371/journal.pgen.0030090)
Supplement: Supplementary file 1 [file pgen.0030090.sg001.pdf]

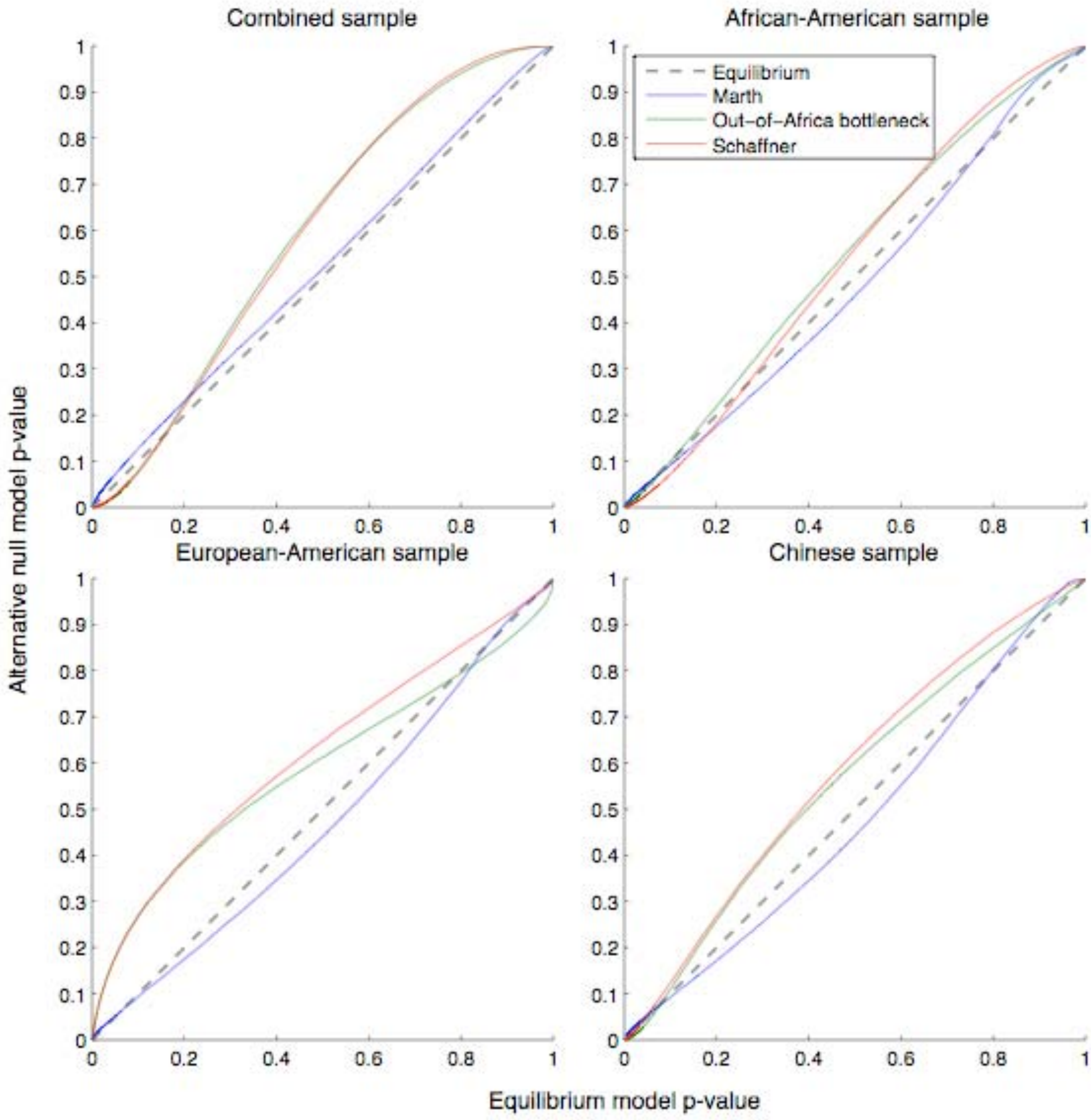
Combined sample
African-American sample
European-American sample
Chinese sample
Equilibrium
Marth
Out-of-Africa bottleneck
Schaffner
Alternative null model p-value
Equilibrium model p-value
0
0.1
0.2
0.3
0.4
0.5
0.6
0.7
0.8
0.9
1
