# Supplementary material for: Localizing Recent Adaptive Evolution in the Human Genome
Source: PLoS Genet. 2007 Jun 1;3(6):e90. doi: 10.1371/journal.pgen.0030090 (PMC1885279; doi:10.1371/journal.pgen.0030090)
Supplement: Supplementary file 2 [file pgen.0030090.st001.doc]

**Table S1.** The 63 genomic regions with strong evidence for a recent selective sweep (*P*<0.00001, CLR test), but where the estimate of the position of the beneficial allele is not within 100kb of the coding sequence of a known gene.

**African-American sample**

| **chr** | **CMLE position** | **CLR** |
| --- | --- | --- |
| 8 | 21060210 | 38.592 |
| 9 | 24692360 | 29.102 |
| 10 | 38009750 | 32.625 |

**European-American sample**

| **chr** | **CMLE position** | **CLR** |
| --- | --- | --- |
| 1 | 186161000 | 47.598 |
| 1 | 235269000 | 32.517 |
| 4 | 33109000 | 39.633 |
| 4 | 112360400 | 40.941 |
| 4 | 166109000 | 33.868 |
| 4 | 172352700 | 43.391 |
| 5 | 109781400 | 36.394 |
| 6 | 95505020 | 34.962 |
| 6 | 124000800 | 32.334 |
| 8 | 16372470 | 34.846 |
| 10 | 6862521 | 44.404 |
| 10 | 60162650 | 36.057 |
| 13 | 20760520 | 32.949 |
| 13 | 31271390 | 32.59 |
| 13 | 36716660 | 36.767 |
| 13 | 62010680 | 45.804 |
| 13 | 103086200 | 37.004 |
| 14 | 43252270 | 40.807 |

**Chinese sample**

| **chr** | **CMLE position** | **CLR** |
| --- | --- | --- |
| 1 | 178419400 | 33.205 |
| 1 | 186167000 | 40.393 |
| 2 | 21929320 | 29.874 |
| 2 | 72478360 | 37.548 |
| 2 | 81652940 | 43.415 |
| 2 | 107665700 | 29.151 |
| 2 | 126867900 | 31.293 |
| 2 | 218116700 | 34.541 |
| 4 | 14355450 | 36.539 |
| 4 | 19874050 | 30.6 |
| 4 | 29030540 | 33.692 |
| 4 | 33096330 | 35.62 |
| 4 | 160914600 | 36.674 |
| 5 | 30107170 | 33.333 |
| 5 | 117599000 | 44.493 |
| 5 | 124550700 | 30.726 |
| 5 | 171974400 | 33.698 |
| 6 | 67178690 | 42.229 |
| 7 | 135264600 | 33.436 |
| 7 | 146839500 | 33.832 |
| 8 | 9328165 | 45.592 |
| 8 | 129501700 | 36.75 |
| 8 | 138877400 | 40.795 |
| 9 | 13908060 | 33.858 |
| 10 | 3921733 | 35.089 |
| 10 | 23905640 | 37.122 |
| 10 | 59082290 | 42.167 |
| 11 | 37922250 | 39.434 |
| 12 | 57702190 | 37.66 |
| 12 | 86646030 | 32.526 |
| 14 | 97031440 | 37.073 |
| 15 | 43952420 | 31.154 |

**Combined sample**

| **chr** | **CMLE position** | **CLR** |
| --- | --- | --- |
| 2 | 82936530 | 37.457 |
| 2 | 195379900 | 24.075 |
| 4 | 33081370 | 33.478 |
| 4 | 182461300 | 24.377 |
| 5 | 162039900 | 24.623 |
| 8 | 23886790 | 28.351 |
| 8 | 36349010 | 36.367 |
| 9 | 9625815 | 26.219 |
| 9 | 24704190 | 23.596 |
| 11 | 38793370 | 37.719 |
| 11 | 108521400 | 24.739 |
| 12 | 86576430 | 26.225 |
| 16 | 75614810 | 24.053 |
| 20 | 20562990 | 25.775 |
