# Supplementary material for: Localizing Recent Adaptive Evolution in the Human Genome
Source: PLoS Genet. 2007 Jun 1;3(6):e90. doi: 10.1371/journal.pgen.0030090 (PMC1885279; doi:10.1371/journal.pgen.0030090)
Supplement: Supplementary file 3 [file pgen.0030090.st003.doc]

**Table S3.** Evidence of selective sweeps at genes involved in the dystrophin protein complex. *P*-values are from the test of the genomic window nearest the midpoint of the gene, and values in parentheses represent the minimum *P*-value for all windows within the gene, which is reported if different from the midpoint *P*-value.

|  |  |  | **CLR P-value** | | | |
| --- | --- | --- | --- | --- | --- | --- |
| **Gene** | **chr** | **position (Mb)** | **Combined** | **Afr. American** | **Eur. Amer.** | **Chinese** |
| DAG1 | 3 | 49518312 | 0.103 | 0.829 | 0.002 | 0.043 |
| DTNA | 18 | 30652312 | 0.00004 (0) | 0.088 (0.08) | 0.0005 (0) | 0.0024 (0) |
| DTNB | 2 | 25713393 | 0.361 | 0.983 (0.62) | 0.384 | 0.195 |
| DTNBP1 | 6 | 15701132 | 0.51 (0.17) | 0.913 (0.42) | 0.644 (0.60) | 0.037 (0.036) |
| SGCA | 17 | 48722738 | 0.072 (0.065) | 0.484 | 0.181 | 0.001 (0.0008) |
| SGCB | 4 | 52812816 | 0.404 | 0.468 | 0.187 | 0.275 |
| SGCD | 5 | 155951580 | 0.021 (0.006) | 0.046 (0.017) | 0.505 (0.18) | 0.033 (0.22) |
| SGCE | 7 | 93862139 | 0.433 | 0.106 | 0.059 | 0.39 |
| SGCG | 13 | 21636257 | 0.268 | 0.047 | 0.997 (0.48) | 0.452 (0.41) |
| SGCZ | 8 | 14190559 | 0.036 | 0.362 (0.09) | 0.057 (0.04) | 0.513 (0.29) |
| SNTA1 | 20 | 32729545 | 0.059 | 0.355 | 0.011 | 0.036 |
| SNTB1 | 8 | 121644190 | 0.401 (-0.17) | 0.769 | 0.715 (0.014) | 0.781 (0.31) |
| SNTB2 | 16 | 69054136 | 0.029 | 0.623 | 0.249 | 0.009 |
| SNTG1 | 8 | 51556054 | 0.213 (0.0002) | 0.599 (0.054) | 0.052 (-0.021) | 0.282 (-0.0003) |
| SNTG2 | 2 | 1065801 | 0.907 | 0.408 | 0.996 | 0.308 |
