# Supplementary material for: Localizing Recent Adaptive Evolution in the Human Genome
Source: PLoS Genet. 2007 Jun 1;3(6):e90. doi: 10.1371/journal.pgen.0030090 (PMC1885279; doi:10.1371/journal.pgen.0030090)
Supplement: Supplementary file 4 [file pgen.0030090.st004.doc]

**Table S4.** Evidence of selective sweeps at heat shock genes. *P*-values are from the test of

the genomic window nearest the midpoint of the gene.

|  |  |  | **CLR *P*-value** | | | |
| --- | --- | --- | --- | --- | --- | --- |
| **Gene** | **chr** | **Position (Mb)** | **Combined** | **Afr. Amer.** | **Eur. Amer.** | **Chinese** |
| DNAJC11 | 1 | 6438343 | 0.627 | 0.613 | **0.005** | 0.314 |
| HSPB7 | 1 | 15705963 | 0.308 | 0.789 | 0.556 | **0.043** |
| DNAJC8 | 1 | 28148335 | 0.096 | 0.779 | **0.004** | **0.003** |
| DNAJC6 | 1 | 65174568 | 0.972 | 0.879 | 0.971 | **0.001** |
| DNAJB4 | 1 | 77898956 | 0.676 | 0.799 | 0.347 | 0.708 |
| HSPA6 | 1 | 158764190 | **0.002** | 0.3 | **0.0002** | **0.00004** |
| DNAJC5G | 2 | 27475875 | 0.907 | 0.968 | 0.405 | 0.546 |
| DNAJC10 | 2 | 183814880 | 0.958 | 0.554 | 0.411 | 0.435 |
| HSPD1 | 2 | 198560210 | 0.972 | 0.255 | 0.239 | 0.186 |
| HSPE1 | 2 | 198569120 | 0.972 | 0.255 | 0.239 | 0.186 |
| DNAJB2 | 2 | 220349670 | 0.802 | 0.874 | 0.211 | 0.16 |
| HSPBAP1 | 3 | 123806760 | 0.316 | 0.717 | **0.047** | 0.119 |
| DNAJB8 | 3 | 129502650 | 0.544 | 0.799 | 0.371 | 0.724 |
| DNAJC13 | 3 | 133524330 | 0.422 | 0.504 | 0.859 | 0.228 |
| DNAJB11 | 3 | 187616860 | 0.592 | 0.789 | 0.514 | **0.002** |
| HSP90BB | 4 | 13088365 | **0.0002** | 0.268 | **0.008** | **0.00004** |
| HSPA4L | 4 | 129187690 | 0.078 | 0.211 | 0.179 | 0.715 |
| DNAJA5 | 5 | 34987742 | 0.331 | 0.119 | 0.379 | 0.337 |
| HSPB3 | 5 | 53767731 | 0.773 | 0.917 | 0.541 | 0.435 |
| HSPA4 | 5 | 132490250 | 0.898 | 0.392 | 0.804 | 0.808 |
| HSPA9B | 5 | 137977580 | 0.968 | 0.957 | 0.971 | **0.0003** |
| HSPA1L | 6 | 31883074 | 0.759 | 0.781 | 0.876 | 0.604 |
| HSPA1A | 6 | 31888783 | 0.759 | 0.781 | 0.876 | 0.604 |
| HSPA1B | 6 | 31900982 | 0.748 | 0.779 | 0.84 | 0.496 |
| HSPCB | 6 | 44265706 | 0.948 | 0.504 | 0.084 | 0.368 |
| HSPB1 | 7 | 75544849 | 0.639 | 0.808 | 0.263 | 0.405 |
| DNAJB9 | 7 | 107773520 | 0.488 | 0.853 | 0.06 | 0.2 |
| DNAJB6 | 7 | 156581660 | 0.675 | 0.543 | 0.338 | 0.143 |
| DNAJC5B | 8 | 67037985 | 0.296 | 0.362 | **0.008** | **0.0005** |
| DNAJA1 | 9 | 33022692 | 0.158 | 0.624 | **0.004** | 0.39 |
| DNAJB5 | 9 | 34985243 | **0.023** | 0.138 | 0.1 | 0.619 |
| HSPA5 | 9 | 123377180 | 0.663 | 0.818 | 0.434 | 0.111 |
| HSPA14 | 10 | 14901009 | **0.002** | 0.543 | **0.005** | **0.006** |
| DNAJC1 | 10 | 22172995 | 0.487 | **0.021** | **0.008** | 0.852 |
| DNAJC12 | 10 | 68921924 | 0.813 | 0.799 | 0.159 | **0.042** |
| DNAJB12 | 10 | 73449714 | 0.158 | 0.624 | **0.004** | 0.39 |
| DNAJC9 | 10 | 74349655 | 0.719 | 0.434 | **0.00003** | **0.021** |
| HSPA5BP1 | 11 | 60473602 | **0.009** | 0.054 | **0.017** | **0.00003** |
| DNAJC4 | 11 | 63775453 | 0.556 | 0.341 | 0.777 | 0.092 |
| SERPINH1 | 11 | 75006569 | **0.002** | 0.334 | **0.001** | **0.034** |
| HSPB2 | 11 | 111321740 | 0.325 | 0.348 | 0.187 | **0.001** |
| HSPA8 | 11 | 122467890 | 0.568 | 0.562 | 0.913 | 0.724 |
| DNAJC14 | 12 | 54505368 | **0.024** | 0.089 | **0.044** | **0.00001** |
| HSPB8 | 12 | 118036340 | 0.057 | 0.101 | 0.338 | 0.477 |
| HSPH1 | 13 | 29521587 | 0.792 | 0.972 | 0.419 | 0.724 |
| DNAJD1 | 13 | 41437573 | **0.005** | 0.151 | 0.343 | 0.3 |
| DNAJC3 | 13 | 94084398 | 0.872 | 0.665 | 0.68 | 0.583 |
| HSP90Bf | 13 | 95237083 | 0.532 | 0.502 | 0.484 | 0.631 |
| HSPA2 | 14 | 62998568 | **0.002** | 0.45 | 0.199 | **0.005** |
| HSPCA | 14 | 100566940 | 0.113 | 0.246 | 0.564 | **0.009** |
| DNAJA4 | 15 | 76280773 | 0.917 | 0.77 | 0.794 | 0.868 |
| DNAJA3 | 16 | 4490692 | 0.218 | 0.738 | 0.398 | 0.29 |
| DNAJA2 | 16 | 46780300 | **0** | 0.287 | **0** | **0** |
| DNAJC7 | 17 | 40522242 | 0.391 | 0.643 | 0.813 | **0.001** |
| HSPB9 | 17 | 40648273 | 0.533 | 0.675 | 0.769 | **0.002** |
| DNAJB1 | 19 | 14488956 | 0.942 | 0.748 | 0.955 | 0.564 |
| HSPB6 | 19 | 40939006 | 0.722 | 0.789 | 0.357 | 0.676 |
| HSPA12B | 20 | 3721067 | **0.002** | 0.613 | 0.581 | **0.00005** |
| DNAJC5 | 20 | 63287658 | 0.898 | 0.643 | 0.652 | 0.053 |
| DNAJB7 | 22 | 39500572 | **0.012** | 0.572 | 0.14 | **0.004** |
