# Supplementary material for: Localizing Recent Adaptive Evolution in the Human Genome
Source: PLoS Genet. 2007 Jun 1;3(6):e90. doi: 10.1371/journal.pgen.0030090 (PMC1885279; doi:10.1371/journal.pgen.0030090)
Supplement: Supplementary file 5 [file pgen.0030090.st005.doc]

**Table S5.** Contingency table analyses for enrichment of significant results in windows

nearest the midpoint of known genes, compared with the remainder of the genome.

Different rows repeat the analysis for different CLR test significance levels (indicated in

parentheses) and for different population samples. For the CLR test in the European-

American and Chinese samples, we observe a highly significant enrichment of CLR tests

that reject the null at gene centers, and this signal becomes stronger with more stringent

significance levels.

|  | **At gene centers** | | **Remainder of genome** | |  |  |
| --- | --- | --- | --- | --- | --- | --- |
| **Test** | **Significant** | **n.s.** | **Significant** | **n.s.** | **χ^2^ (1 d.f.)** | **P-value** |
| Combined  (*P*<0.05) | 3175 | 14309 | 16288 | 77023 | 5.038 | **0.012** |
| Combined  (*P*<0.01) | 1608 | 15876 | 8332 | 84979 | 1.292 | 0.128 |
| Combined  (*P*<0.001) | 642 | 16842 | 3433 | 89878 | 0.002 | 0.518 |
|  |  |  |  |  |  |  |
| Afr.-Amer  (*P*<0.05) | 1015 | 16469 | 6422 | 86889 | 27.279 | 1 |
| Afr.-Amer  (*P*<0.01) | 356 | 17128 | 2307 | 91004 | 11.945 | 0.9997 |
| Afr.-Amer  (*P*<0.001) | 134 | 17350 | 682 | 92629 | 0.2542 | 0.307 |
|  |  |  |  |  |  |  |
| Eur.-  Amer.  (*P*<0.05) | 3367 | 14117 | 17282 | 76029 | 5.271 | **0.011** |
| Eur.-  Amer.  (*P*<0.01) | 1940 | 15544 | 8488 | 84823 | 69.04 | **5.6X10^-17^** |
| Eur.-  Amer.  (*P*<0.001) | 763 | 16721 | 2765 | 90546 | 93.722 | **0** |
|  |  |  |  |  |  |  |
| Chinese  (*P*<0.05) | 4068 | 13416 | 20824 | 72487 | 7.633 | **0.0029** |
| Chinese  (*P*<0.01) | 2261 | 15223 | 11130 | 82181 | 13.968 | **9.3 x 10^-5^** |
| Chinese  (P<0.0001) | 947 | 16537 | 4470 | 88841 | 12.407 | **0.00021** |
