# Supplementary material for: Localizing Recent Adaptive Evolution in the Human Genome
Source: PLoS Genet. 2007 Jun 1;3(6):e90. doi: 10.1371/journal.pgen.0030090 (PMC1885279; doi:10.1371/journal.pgen.0030090)
Supplement: Supplementary file 6 [file pgen.0030090.st006.doc]

**Table S6.** Evidence of a selective sweep by the CLR test in the most extreme genomic regions identified by other methods in the Hapmap analysis. Values in parentheses indicate *P*-values of the CLR statistic.

| **population differentiation (Fst)** | | |  |  |  |  |
| --- | --- | --- | --- | --- | --- | --- |
| chr. | location (Mb) | gene | Combined CLR | Afr.-Amer. CLR | Eur.-Amer. CLR | Chinese CLR |
| 1 | 54.77 | *THEA* | **10.824 (0.0247)** | 4.8008 (0.6129) | 8.0791 (0.234) | **20.1831 (0.0007)** |
| 1 | 156 | *FY* | **17.3896 (0.0002)** | 3.2234 (0.8905) | **14.8759 (0.0141)** | **19.214 (0.0012)** |
| 2 | 3.18 | *COLEC11* | 6.3252 (0.371 | 5.2956 (0.5231) | 9.2255 (0.1554) | 6.2749 (0.4126) |
| 2 | 73.59 | *ALMS1* | 3.6585 (0.8982 | 2.4582 (0.9764) | 2.9553 (0.9322) | 6.6304 (0.3679) |
| 2 | 109 | *EDAR* | **15.1893 (0.0033)** | 2.9832 (0.8841) | **30.4703 (0.0001)** | **40.0346 (0)** |
| 3 | 182 | *FXR1* | 10.1446 (0.0397) | 9.1239 (0.1138) | 10.882 (0.0799) | 9.1926 (0.1371) |
| 3 | 185 | *MCF212* | 8.5891 (0.1062 | 6.3671 (0.3546) | 9.7885 (0.1219) | 4.6536 (0.667) |
| 4 | 41.84 | *SLC30A9* | **11.0551 (0.0204)** | 6.6847 (0.3201) | 7.848 (0.2584) | **38.2665 (0)** |
| 4 | 46.57 | *ENSG* | **12.9438 (0.0117)** | **13.1433 (0.0333)** | 12.6599 (0.0544) | 8.6286 (0.1993) |
| 4 | 101 | *ADH1B* | 5.2366 (0.5915) | 6.5958 (0.3268) | 6.5648 (0.3977) | **24.6107 (0.0001)** |
| 8 | 10.52 | *RP1L1* | 7.0252 (0.2718) | 3.1211 (0.8909) | 4.6967 (0.6632) | 7.0462 (0.3195) |
| 10 | 50.4 | *ERCC6* | 5.1491 (0.6027) | 8.3756 (0.1589) | 4.7904 (0.6612) | 4.8409 (0.6387) |
| 10 | 71 | *NEUROG3* | 5.2118 (0.5915) | 3.9422 (0.7685) | 4.664 (0.6796) | 6.7715 (0.3468) |
| 11 | 46.7 | *F2* | 4.888 (0.6627) | 3.8302 (0.7886) | 8.9431 (0.1723) | 6.20006 (0.4204) |
| 15 | 46.21 | *SLC24A5* | 4.3127 (0.7917) | 6.9089 (0.2931) | **22.5088 (0.0005)** | 3.3663 (0.8841) |
| 15 | 61.72 | *HERC1* | 3.8653 (0.8496) | 3.761 (0.7738) | 5.4118 (0.5444) | **31.2198 (0)** |
| 16 | 31 | *ZNF646* | 9.0434 (0.087) | 3.1745 (0.8892) | **14.0609 (0.0231)** | 7.3945 (0.2829) |
| 16 | 46.82 | *ABCC11* | **23.4455 (0)** | 6.9236 (0.2865) | **49.0259 (0)** | **58.4467 (0)** |
| 17 | 26.32 | *RNF135* | 4.7007 (0.6986) | 7.158 (0.2624) | 7.2839 (0.3128) | 5.3669 (0.5465) |
| 18 | 66.02 | *RTTN* | 6.7075 (0.3288) | 2.3003 (0.9677) | **25.5272 (0.0006)** | **19.6591 (0.0028)** |
| 19 | 5.78 | *FUT6* | **14.3181 (0.002)** | 5.06 (0.5619) | **12.6768 (0.037)** | **17.649 (0.0026)** |
| 19 | 47.72 | *CEACAM1* | **11.1469 (0.0195)** | 8.6441 (0.1408) | **29.2804 (0)** | **13.1231 (0.0238)** |
| 22 | 18.16 | *GNB1L* | 4.2522 (0.8023) | 5.5158 (0.486) | 3.2889 (0.8917) | 6.003 (0.4516) |
|  |  |  |  |  |  |  |
| **EHH** |  |  |  |  |  |  |
| chr | location (Mb) | population | nearest gene | CLR |  |  |
| 2 | 137.22 | Eur. | *CXCR4* | **18.7309 (0.004)** |  |  |
| 5 | 22.3 | Afr. | *IRX4* | 7.8695 (0.1964) |  |  |
| 7 | 79.9 | Afr. | *CD36* | **11.9375 (0.0474)** |  |  |
| 7 | 73.75 | Eur. | *PMS2L5* | **15.0025 (0.0135)** |  |  |
| 12 | 109.89 | Eur. | *CUTL2* | 10.447 (0.0932) |  |  |
| 15 | 78.56 | Afr. | *ARNT2* | 9.1349 (0.1138) |  |  |
| 16 | 75.66 | Afr. | *TERF2IP* | **15.8499 (0.0048)** |  |  |
| 17 | 3.95 | Afr. | *CAMKK1* | 5.7262 (0.4501) |  |  |
| 18 | 24.5 | Eur. | *CDH2* | 5.3094 (0.5484) |  |  |
| 22 | 32.46 | Afr. | *LARGE* | 4.3917 (0.6537) |  |  |
